# An uncommon cause of inappropriate ICD shock

**DOI:** 10.1002/ccr3.2782

**Published:** 2020-03-10

**Authors:** George Bazoukis, Konstantinos P. Letsas, Konstantinos Vlachos, Gary Tse, Dimitrios Manolatos, Antonios Sideris, Michael Efremidis, Sotirios Xydonas

**Affiliations:** ^1^ Second Department of Cardiology Evangelismos General Hospital of Athens Athens Greece; ^2^ Tianjin Key Laboratory of Ionic‐Molecular Function of Cardiovascular Disease Department of Cardiology, Tianjin Institute of Cardiology Second Hospital of Tianjin Medical University Tianjin China; ^3^ Xiamen Cardiovascular Hospital Xiamen University Xiamen China

**Keywords:** electromagnetic interference, implantable cardioverter‐defibrillator, inappropriate interventions, inappropriate shock

## Abstract

In cases of electromagnetic interference (EMI), if the source of the inappropriate EMI cannot be identified, then the sensitivity of the device could be decreased, or the cycle length of the VF detection trigger zone changed.

## Graphical abstract

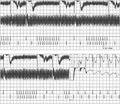

## 1 CLINICAL IMAGE

**Figure 1:**
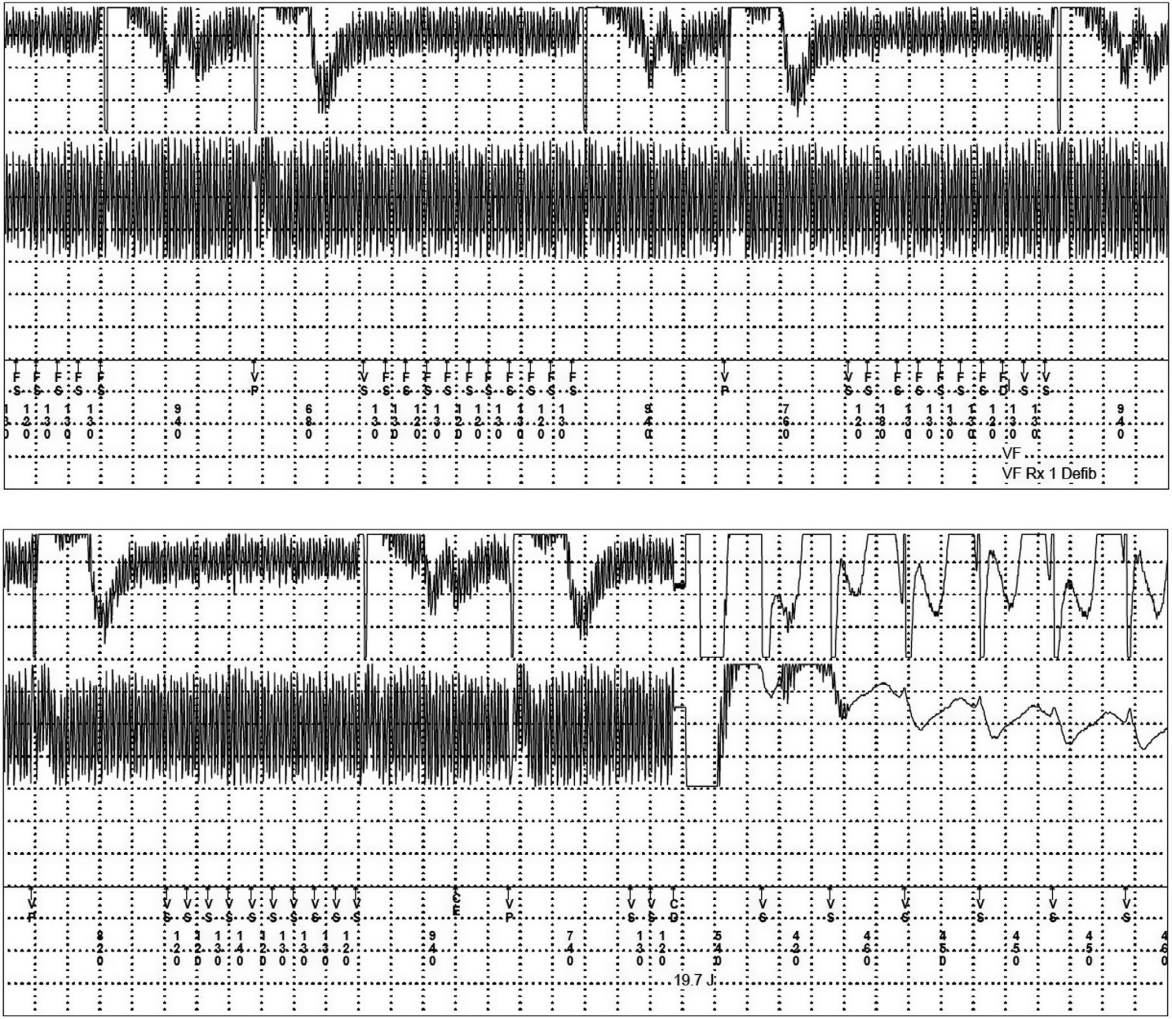
Stored intracardiac electrograms recorded during this event showed high‐frequency undulating noise. This was interpreted by ICD as VF and was shocked with 19.7J

The patient is a 74‐years‐old male with a history of ischemic cardiomyopathy with ejection fraction 30%, prosthetic aortic valve, chronic kidney disease, and an implantable cardioverter‐defibrillator (ICD) (Maximo II VR D284VRC) implanted for primary prevention of sudden cardiac death. The patient was admitted at the emergency department because he received an ICD shock while he was taking a shower. The device was programed to detect ventricular fibrillation (VF) at >176 bpm. Device interrogation retrieved the following electrograms (Figure 1). What's your diagnosis?

## 2 ANSWER

During the episode, the patient reported that he was taking a shower while the water heater was on. The restroom was examined by a certified electrician who found a small leak into the bath. As a result, the shock was classified as inappropriate caused by electromagnetic interference after excluding lead and device problem. Ventricular oversensing is the condition in which pacemakers and implantable cardioverter‐defibrillators (ICDs) sense signals that do not represent local depolarizations. Electromagnetic interference is a well‐known cause of inappropriate shocks, and several cases have been reported.^1^, ^2^ In most cases, ventricular oversensing episodes can be prevented by appropriate ICD reprograming, lead replacement, or avoidance of the initiating trigger.

## CONFLICTS OF INTEREST

The authors declare no conflicts of interest.

## AUTHORS’ CONTRIBUTION

GB: wrote the first draft and contributed to the management of the patient and approval of the submitted manuscript; KPL: contributed to the management of the patient, major revision, and approval of the submitted manuscript; KV: contributed to the management of the patient, major revision, and approval of the submitted manuscript; GT: contributed to major revision and approval of the submitted manuscript; DM: contributed to the management of the patient, major revision, and approval of the submitted manuscript; AS: contributed to the management of the patient, major revision, and approval of the submitted manuscript; ME: contributed to the management of the patient, major revision, and approval of the submitted manuscript; SX: contributed to the management of the patient, major revision, and approval of the submitted manuscript.
